# Application of Taro (*Colocasia esculenta*) Mucilage as a Promising Antimicrobial Agent to Extend the Shelf Life of Fresh-Cut Brinjals (Eggplants)

**DOI:** 10.3390/gels9110904

**Published:** 2023-11-15

**Authors:** Mansuri M. Tosif, Aarti Bains, Gulden Goksen, Nemat Ali, Alexandru Vasile Rusu, Monica Trif, Prince Chawla

**Affiliations:** 1 Department of Food Technology and Nutrition, Lovely Professional University, Phagwara 144411, Punjab, India; tosifmansuri444@gmail.com; 2 Department of Microbiology, Lovely Professional University, Phagwara 144411, Punjab, India; aarti05888@gmail.com; 3 Department of Food Technology, Vocational School of Technical Sciences at Mersin Tarsus Organized Industrial Zone, Tarsus University, 33100 Mersin, Türkiye; guldengoksen@tarsus.edu.tr; 4 Department of Pharmacology and Toxicology, College of Pharmacy, King Saud University, P.O. Box 2457, Riyadh 11451, Saudi Arabia; nali1@ksu.edu.sa; 5 *CENCIRA* Agrofood Research and Innovation Centre, Ion Meșter 6, 400650 Cluj-Napoca, Romania; 6 Centre for Innovative Process Engineering (CENTIV) GmbH, 28857 Syke, Germany; monica_trif@hotmail.com

**Keywords:** anti-microbial, mucilage, polysaccharide, edible coatings, gel forming

## Abstract

Taro rhizomes are a rich source of polysaccharides, including starch and mucilage. However, mucilage has excellent anti-microbial efficacy, and unique gel-forming and techno-functional properties. Therefore, this study aimed to extract and utilize taro mucilage (TM), which is viscous and has a gel-like texture, for the shelf-life enhancement of fresh-cut brinjals (eggplants). Mucilage was extracted using hot-water extraction and the yield was calculated to be 6.25 ± 0.87% on a dry basis. Different formulations of coating gel solutions were prepared: 1, 2, 3, 4, 5, 6, and 7%. The selection of the coating gel solution was carried out based on particle size. The smallest particle size was observed in treatment T5 (154 ± 0.81 nm) and zeta potential −27.22 ± 0.75 mV. Furthermore, cut brinjals were coated with the prepared mucilage gel solution and this showed a significant effect on the overall physicochemical properties of cut brinjals. Maximum weight loss occurred on the 10th day (12.67 ± 0.24%), as compared with coated brinjals (8.99 ± 0.42%). Minor changes were observed in pH, for the control sample significantly decreased from 4.58 ± 0.45 to 2.99 ± 0.75 on the 0th day to the 10th day, respectively. Titrable acidity of coated and uncoated cut brinjals was found to be at 0.31 ± 0.44% on the 0th day, which increased up to 0.66 ± 0.20% for the control and 0.55 ± 0.68% for coated brinjals on the 10th day. The taro mucilage coating gel (TMCG) solution showed pseudo-plastic behavior or shear-thinning fluid behavior. FTIR data confirmed the existence of several functional groups including various sugars, proteins, and hydroxylic groups. Antioxidant activity of coated and uncoated cut brinjals was found to be 22.33 ± 0.37% and 22.15 ± 0.49%, respectively. The TMCG solution showed effective results towards the various food pathogenic microorganisms. Overall, it is a natural, renewable resource that is biodegradable. This makes it an environmentally friendly alternative to synthetic additives or thickeners. It is cost effective, easily available, eco-friendly, and non-toxic. This can be an attractive feature for consumers looking for sustainable and eco-friendly options.

## 1. Introduction

Fresh fruits and vegetables are rich in beneficial health-promoting nutrients and are consumed and produced in abundance worldwide. However, these foods are highly sensitive to environmental factors including temperature, microorganisms, humidity, and oxygen [1,2]. Therefore, the storability of fruits and vegetables at ambient temperature has been a significant challenge for researchers. Moreover, several other foods are often consumed without any processing or cooking treatment. Preservation and maintenance of food quality is crucial to the consumers as well as to food industries, due to the dependency of market demand and acceptance, ultimately affecting the economic value [3,4]. In this context, several innovations in the packaging material, edible packaging, active packaging, controlled atmosphere storage, modified atmosphere packaging, and cold-distribution chain have been employed for the enhancement of the shelf life of food products. Edible coating and packaging have gained significant attention, primarily due to their sustainability, affordability, convenience, and ease of industrial and commercial applications [5]. Furthermore, they align with the growing consumer preference for food that ensures safety and contains fewer synthetic additives [6]. Edible coatings can be well-defined as the barrier layer applied on the surface of food to wrap it, with the multi-purpose of extending the shelf life of food products. As consumer interest in plant-based products is increasing, there is a demanding need to minimize the reliance on chemical-based additives in the postharvest treatment of food. While a variety of chemical compounds are employed to prolong the shelf life of fruits and vegetables, some of them show adverse effects on human health and are unsafe for the environment. Additionally, the use of fungicides and synthetic substances has contributed to the emergence of pathogen-resistant strains with progressively higher levels of toxic residues [7].

Mucilage is a water-soluble, edible hydrocolloid extracted from various sources of plants, animals, and bacteria. It is famous due to its outstanding techno-functional importance and therapeutical properties [8]. Therefore, mucilage is potentially useful in the food, pharmaceutical, textile, and paper industries. In addition, it is a good natural alternative source of anti-microbial agents, which increases its demand in various fields of research. Taro (*Colocasia esculenta*) are rhizomes categorized as a member of the Araceae family and hold substantial importance as a demanding crop in the human diet, primarily thriving in tropical and humid regions across the world [9,10]. Mucilage is a gel type of viscous material that contains proteins and polysaccharides, and it has excellent water-holding capacity due to the presence of hydroxylic groups. Mucilage extracted from plant sources has a good water-holding capacity and unique gel-forming property due to the existence of monopolar compounds; thus, it can hold a large amount of water and, as an edible coating material, can also prevent food from food spoilage. The rheological behavior of mucilage is directly affected by the gelling property of mucilage. Several studies indicate that taro can serve as a valuable source of polysaccharides, including mucilage, ranging from 3 to 19%, and starch, which can account for 70 to 80% of its composition, depending upon the method of extraction. Moreover, the chemical structure of TM includes carbohydrates (together with glucose, galactose, mannose, xylose, and arabinose) and proteins and glycol-protein (mainly consisting of amino acids like leucine, isoleucine, cysteine, tryptophan, and lysine) [11]. Amino acids including tryptophan, isoleucine, and leucine are present and act as hydrophobic portions of radicals. They can enhance the emulsifying characteristics of TM. They also contain substantial expanses of arabinogalactan proteins (AGPs) [12]. Thus, the protein molecules with non-polar radicals within TM are believed to contribute to its hydrophobic characteristics, while polysaccharides play a role in its hydrophilic nature. Furthermore, numerous studies on TM have indicated that starch is a predominant impurity in mucilage, possibly impacting its practical and economic applications [9,11]. The utilization of edible coatings has gained importance, mainly due to their numerous advantages and properties compared with synthetic coatings. Synthetic coatings have contributed significantly to environmental pollution, given their non-biodegradable nature. This environmental concern has raised important considerations for both consumers and governmental bodies, prompting the exploration of alternative solutions. In addition, green extraction approaches for the extraction of TM is a cost-effective and time-consuming method, which attracted the choice and demand of naturally originated material by replacing synthetic polymers [13,14].

Fresh-cut brinjals (eggplants) are highly sensitive to various environmental factors including gases, microbes, and temperature, which result in the spoilage or browning of the brinjals. In this perspective, several synthetic materials or coatings are used for the prevention of brinjals from hazards. Moreover, TM is a cost-effective, naturally anti-microbial, eco-friendly material that possesses excellent gelling properties. However, to date, very few reports have been published on the utilization of TM as a sustainable ingredient. Therefore, the main aim of this research is to explore TM gel as a green coating material for the shelf-life enhancement of fresh-cut brinjals. Thus, to provide the systematic industrial application and characterization of this coating solution on the physico-chemical property relationships of TM gel, this study revisited the coating phenomena of mucilage gel, with a focus on the shelf-life enhancement of fresh-cut brinjals.

## 2. Results and Discussion

### 2.1. Mucilage Yield, Zeta Potential, and Particle Size

Mucilage was extracted using hot-water extraction and the yield of TM was found to be 6.25 ± 0.87% on a dry basis. Mucilage is a water-soluble edible polysaccharide and rhizomes are a rich source of starch. In this context, a few fractions of the starch were observed along with mucilage. However, for the coating applications, there are no limitations on starchy compounds. Therefore, hot-water extraction is a suitable method for the extraction of mucilage due to its higher yield recovery. The zeta potential and particle size of different coating gel solutions are shown in Table 1. The smallest particle size (154 ± 0.81 nm) was observed in treatment T5 with a zeta potential of −27.22 ± 0.75 mV, whereas the higher particle size (224 ± 0.68 nm) of the coating gel solution was observed at treatment T1, which was due to the smaller concentration of mucilage. TM is a viscous substance, along with a starch compound, and it swells in the presence of water due to the existence of hydroxylic groups that interact with the hydrogen atoms of the water resulting in the strong hydrogen bonding between them. Thus, mucilage binds or traps water along with its structure. The average particle size of the solution for treatments T2, T3, T4, T6, and T7 were 204 ± 0.94 nm, 189 ± 0.73, 154 ± 0.81 nm, 166 ± 1.68 nm, and 187 ± 0.47 nm, respectively.

**Figure 1:**
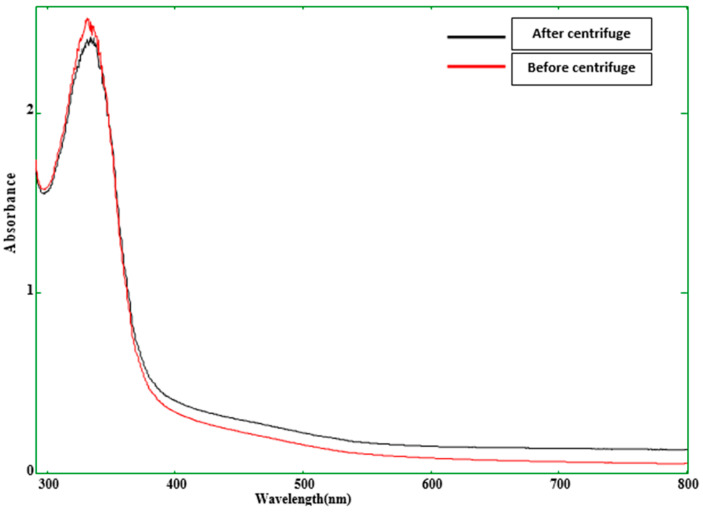
UV visible spectrum of the TM solution containing 1% (*w*/*v*) mucilage.

This trend was observed due to the aggregation of particles in the higher concentration of mucilage in the aqueous medium, whereas, in lower concentrations, it decreased due to the more diluted form of mucilage solution and smaller surface area. Also, the concentration of solid particles of TM, chemical reactions, variation in the surface charge of the mucilage particles, and interaction of mucilage with water may affect the particle size of the coating solution. Wijesena et al. [15] stated that several factors such as temperature and concentration of polysaccharides directly affect the particle size. Herein, it has been observed that with the increasing concentration of the mucilage particle size of the solution increased due to the uniform mixing of the solution at a particular speed. Different concentrations of the mucilage affected the gelling properties of mucilage. Therefore, the particle size and zeta potential of all the treatments are varied. Furthermore, TM powder showed excellent water solubility due to the smaller particles, as shown in Figure 1. Minor variation was observed at the 330 nm wavelength before the centrifuge and after the centrifuge of the TM solution. It proved that TM has remarkable swelling properties when it is mixed with water. The swelling property of TM contributes to its solubility by increasing the surface area exposed to the solvent.

### 2.2. Rheological Behavior of the Coating Gel Solution

**Figure 2:**
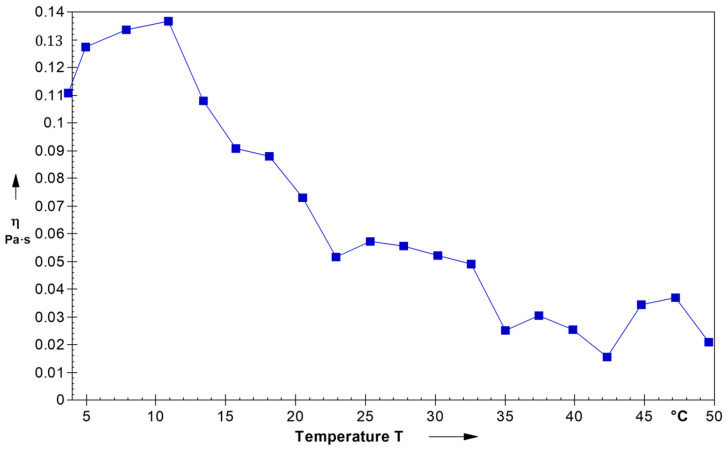
Rheological behavior of TMCG solution.

The swelling capacity and formation of a three-dimensional gel-type network of mucilage and other polysaccharides during the gelation process is a parameter that involves a transition from a liquid (sol) state to a gel state in the presence of heat. This transition is marked by significant changes in viscoelastic properties and the development of solid-like characteristics. Herein, the viscosity effect of temperature on the TMCG solution is shown in Figure 2 for the shear rate 0.14 s^−1^. The TMCG solution showed pseudo-plastic behavior or shear-thinning fluid behavior because the result showed a decrease in viscosity with the increasing shear rate. Moreover, the rheological (viscoelastic) properties of the gelling system are often evaluated to regulate its suitability for commercial applications. Determining the rheological behavior is a key approach to estimating the stability of gels produced through the addition of gums. This is because interactions between different polymers can influence and modify the rheological profile of the gel. Understanding these rheological changes is essential for assessing the efficacy and performance of the gel for its intended commercial uses [11].

### 2.3. Determination of Functional Groups

**Figure 3:**
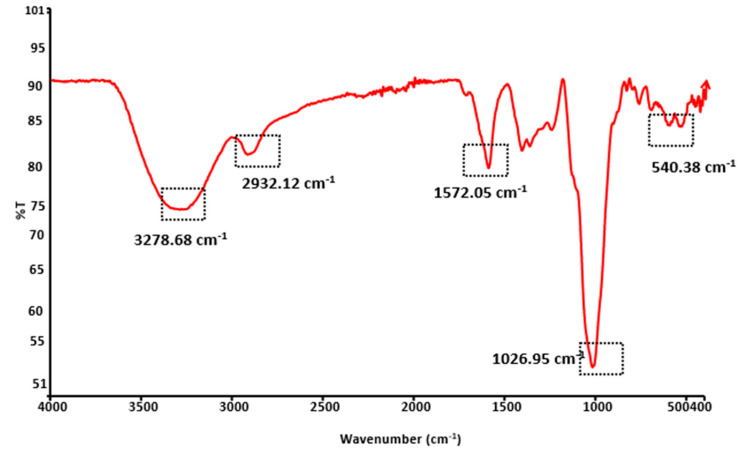
Determination of functional groups in TMCG solution using FTIR.

The existence of several functional groups in the TMCG solution was confirmed by FTIR, and the data are represented in Figure 3. The presence of hydroxylic group (-OH) stretching was confirmed at the wavenumber of 3278.68 cm^−1^. Hydroxylic groups are the major contributor of polysaccharides in their functional properties. Mucilage has an excellent water-holding capacity and gelling ability due to the presence of the hydroxylic group. It has been believed that, when mucilage comes into contact with water (H_2_O), the hydrogel atom of hydrogen and the hydrogen atom of the hydroxylic group create a strong hydrogen bond. Therefore, the gel solution has excellent water and oil binding ability. TM contains a number of proteins (N-H bending) which was confirmed by FTIR at the wavenumber of 1572.05 cm^−1^. The emulsifying ability of mucilage was due to the presence of various sugar and protein molecules. Similar bands were observed in a study performed by Andrade et al. [16]; they extracted mucilage from taro rhizomes using five different methods. However, FTIR data confirmed the presence of protein molecules in the starchless mucilage sample, which was directly interconnected with the functional properties of mucilage. They observed the band at 2962 cm^−1^, which was attributed to the axis deformation of the C-H bond found in the region between 3000 and 2840 cm^−1^. In our study, a similar band was observed at the wavenumber 2932.12 cm^−1^. Arabinose and Galactose is the major sugar existing in the TMCG solution. This was confirmed at the wavenumber of 1026.95 cm^−1^, which was attributed to C-O vibration. Likewise, all peaks owe their significance to the structural and functional properties of mucilage.

### 2.4. Scanning Electron Microscopy (SEM) of TMCG

**Figure 4:**
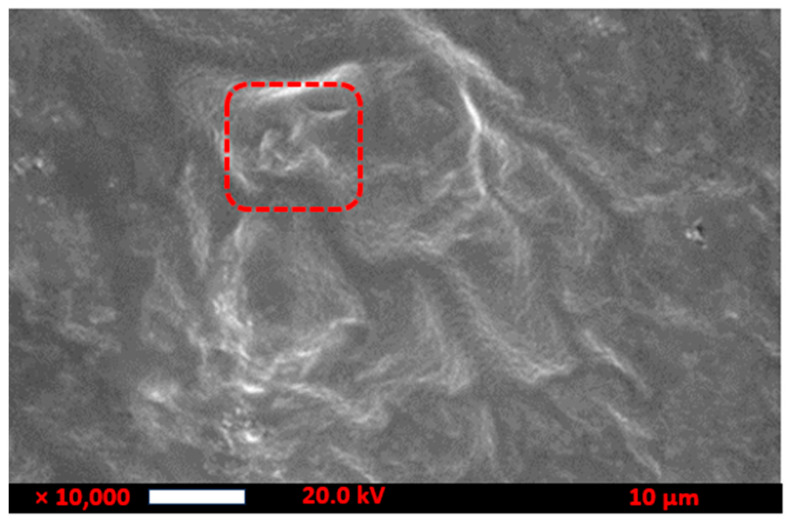
Scanning electron microscopy (SEM) of the TMCG.

The prepared TMCG was analyzed by the SEM at 10,000 magnification to evaluate its surface morphology. TMCG were found to be uniform in shape, spongy, porous, and spherical structure, as shown in Figure 4. The porosity of TMCG helps for the entry and release of the water. Several pores were present on the surface, which was attributed to the presence of the hydrophilic nature of TM. Imaging of the gel surface was observed to rapidly induce ablation of the gel water content. These findings are well supported by a study conducted by Thakur et al. [17] who developed the gum Arabic/guar gum-based biopolymeric nano hydrogel for the enhancement of the shelf life of the grapes and dye reduction. In their study, SEM results revealed that irregular and asymmetrical shapes were observed for the embedded nano hydrogel with the metal oxide nanoparticles. Furthermore, similar morphological features (smooth and even surface) were found for the control (gum Arabic/guar gum-based nano hydrogel. The incorporation of nanomaterial (metal oxide nanoparticles) into the nano hydrogel showed the presence of radiance polygonal and tiny shapes.

### 2.5. Physicochemical Properties of Edible Coated Cut Brinjals

**Figure 5:**
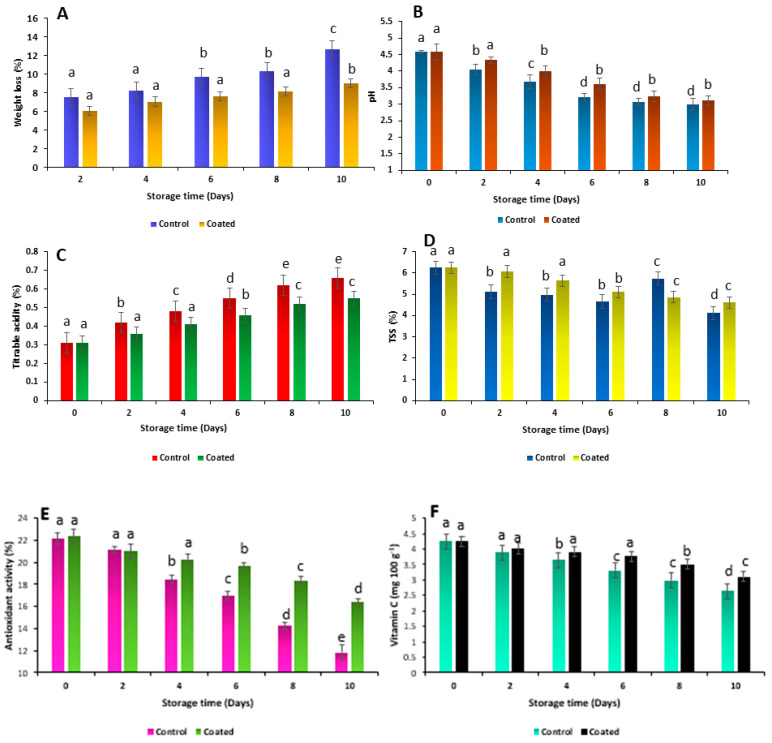
Physicochemical properties of coated and uncoated brinjals: (**A**) weight loss, (**B**) pH, (**C**) titrable acidity, (**D**) TSS, (**E**) antioxidant activity, (**F**) vitamin C (the error bars represent the means with standard error of the two replicate samples, and various lowercase letters show the significant difference (*p* ≤ 0.05) between coating treatments).

An effect of TM edible coating on weight loss and pH is shown in Figure 5A and Figure 5B, respectively. Herein, the weight loss of the control brinjals significantly decreased as compared with the coated sample during the 10-day storage period. Water activity, moisture content, and respiration rate are the major factors that directly affect the physicochemical properties of fruits and vegetables. Maximum weight loss occurred on the 10th day (12.67 ± 0.24%) as compared with coated brinjals (8.99 ± 0.42%). These results proved that TM as an edible coating material created a barrier on the surface of cut brinjals which protects them from environmental factors. A similar observation was found in our previous study by Tosif et al. [18]. In this study, an aloe vera and carboxymethyl cellulose blend edible coating reduced the weight loss of cut apples and effectively enhanced the shelf life of the food product by preventing them from intrinsic and extrinsic factors. In this context, TMCG can serve as a barrier to various gases including carbon dioxide and oxygen. These gases play an important role in the respiration rate of the brinjals by limiting the exchange of gases between the food product and the surrounding environment. Therefore, TMCG reduced the weight loss associated with the respiration rate with gas exchange [7], whereas the pH of the control sample significantly decreased from 4.58 ± 0.45 to 2.99 ± 0.75 on the 0th day to the 10th day, respectively. Likewise, a minor change in pH was observed for TMCG-coated brinjals, which decreased from 4.58 ± 0.25 to 3.11 ± 0.46 on the 0 days to the 10th day, respectively. The pH of a food product plays an important role in influencing the growth of bacteria, yeasts, and molds. Microbial growth tends to be thwarted by extremely low or high pH values depending upon the nature of the microorganism. In practical terms, it is rare to find unprocessed foods with pH levels high enough to provide significant preservation benefits. Various foods do possess pH levels that offer some degree of protection against microbial growth. Nevertheless, only a limited number of foods exhibit pH levels low enough to entirely inhibit the proliferation of microorganisms, particularly yeasts and molds, which can thrive in environments with lower pH levels compared with most bacteria. For the preservation of almost all food items, a combination of microbiological control measures is necessary. These decreases in pH may be due to the activity of some microorganisms and the conversion of sugar to alcohols and acids or some electrolyte sedimentation during storage. Amanulla et al. [19] prepared the aloe vera edible coating for the enhancement of eggplants and studied the effect of aloe vera as a coating material at different storage conditions. The obtained results showed that weight loss, shriveling, total soluble solids, pH, and sugar (total sugar and non-reducing sugar) increased, and firmness, stem color, acidity, reducing sugar, and vitamin C were minimized during the storage period. The 0.5% aloe vera coating at 10 °C showed a significant effect and delayed the changes in the above parameters. The aloe vera coating delayed the changes in the TSS of eggplants, whereas the TSS of uncoated eggplants was increased slowly during the storage period. The TSS of coated eggplants kept at 10 °C was significantly less increased than that of coated eggplants kept at (30 ± 2) °C.

This typically includes methods such as heat processing, refrigeration, freezing, or drying. Among these, canning or heat processing stands out as one of the most widely utilized techniques for food preservation. Titrable acidity and TSS are key parameters to evaluate the overall quality of food products. Results of titrable acidity and the TSS of coated and uncoated brinjals are shown in Figure 5C and 5D, respectively. This shows that a low increase in TSS during storage in treated brinjals was due to slow weight loss that caused less dehydration of brinjals. Another reason there might be an increase in TSS is because of the conversion of complex sugars into simple sugars. Titrable acidity of coated and uncoated cut brinjals was to be found at 0.31 ± 0.44% on the 0th day, which was increased up to 0.66 ± 0.20% for the control and 0.55 ± 0.68% for coated ones on the 10th day. It has been observed that TMCG-coated cut brinjals showed remarkable performance on the overall physicochemical properties of the brinjals. Titratable acidity, also referred to as total acidity, quantifies the total acid content in the brinjal coated and control samples. It was calculated through a thorough titration process where the inherent acids in the food are neutralized using a standard base solution. Titratable acidity proves to be a more accurate indicator of how acidity influences flavor compared with pH measurements. Gel itself creates a protective barrier on the surface of food and prevents several environmental factors. Furthermore, the TSS of the coated and uncoated cut brinjals were found to be at 6.25 ± 0.28% on the 0th day, which significantly decreased to 4.12 ± 0.11% for the control and 4.61 ± 0.69% for coated cut brinjals. The TSS is the most significant factor of brinjals for determining their taste and maturity. TSS content also varies depending on the cultivator. Brinjals mainly contain acids, sugars, vitamins, phenolics, and minerals. Anjum et al. [20] prepared the edible coating solution from the gum Arabic and aloe vera blend for the shelf-life enhancement of guava. In this study, fruits of garlic extract and gum Arabic treatment had higher titratable acidity, and this combination also suppressed the excessive increase in total soluble solids until the end of the storage period compared with the control. Therefore, more research is needed on the incorporation of plant-based ingredients with TMCG.

Results of the antioxidant activity and ascorbic acid are highlighted in Figure 5E and Figure 5F, respectively. Antioxidant activity of coated and uncoated brinjals on the 0th day was found to be 22.33 ± 0.37% and 22.15 ± 0.49%, respectively. Antioxidant activity of the control and coated brinjals significantly decreased on the 10th day up to 11.81 ± 0.67% and 16.36 ± 0.58%, respectively. Ascorbic acid (vitamin C) and antioxidants might help in preventing free radical damage to cells. Lignans, phenols, tannins, and flavonoids are additional naturally occurring antioxidants. The richest sources are foods made from plants [17]. A similar trend was observed in a study carried out by Singh et al. [21]. They prepared the edible coating gel solution from carnauba wax and enhanced the shelf life of cut eggplants (brinjals). Maximum antioxidant activity (67.63 and 51.52 mmol Trolox equivalent antioxidant capacity (TEAC)/100 g FW) was observed in the edible coating of carnauba wax and minimum antioxidant activity (23.61 and 20.60 mmol TEAC/100 g FW) was observed in the control eggplants, packaged and unpackaged, respectively, after 12 days. Consequently, Rangaraj et al. [22] prepared blend composite films from the silver–sepiolite-hybrid-reinforced active gelatin/date waste extract for food packaging application. Results revealed that combining the antioxidant additive and the antibacterial agent into the edible film matrix is one of the most effective strategies to produce active bio-composite film with enriched food packaging properties. Moreover, gelatin/date waste film showed the highest DPPH inhibition activity of 91% and 58% at the end of 6 h in the aqueous and fatty medium, respectively.

**Figure 6:**
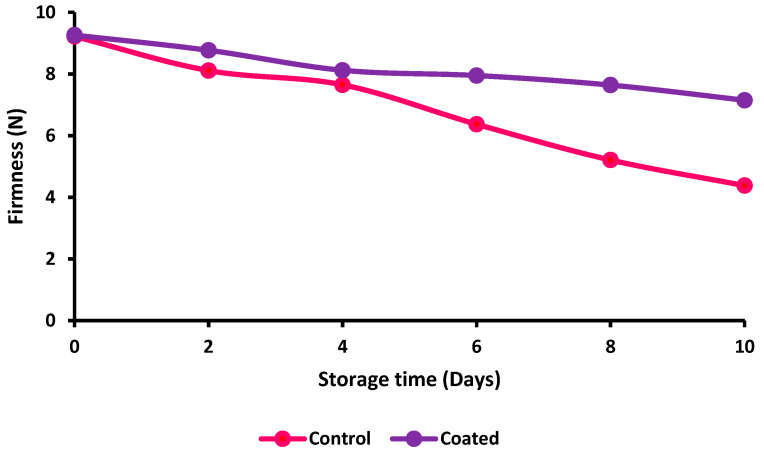
Firmness of coated and uncoated cut brinjals.

The total vitamin C content of the coated and uncoated brinjals was 4.25 ± 0.27% on the 0th day, which significantly decreased up to 2.64 ± 0.69% for the control and 3.11 ± 0.73% for coated brinjals. In this perspective, it has been believed that higher-ascorbic-content-containing foods can help to maintain cartilage, bones, blood vessels, and healthy skin. A similar observation was found in a study performed by Singh et al. [21]. The vitamin C content of coated samples was retained as compared with the control sample. The firmness of the control and coated brinjals is shown in Figure 6. The firmness of the control cut brinjals and coated brinjals was found to be 9.22 ± 0.15 N and 9.26 ± 0.37 N on the 0th day, which significantly decreased up to 3.38 ± 0.71 N and 7.15 ± 0.34 N, respectively. Firmness is a crucial factor in determining the time to harvest, the maturity of the fruit or vegetable, and the quality level of the fruit or vegetable. Brinjal firmness measurement is a lengthy and challenging task. This is certainly applicable to fruits whose hue has no significant connection regardless of the hardness of the brinjals [22]. Likewise, a minimum decrease (8.56 N–6.92 N and 8.56 N–5.63 N) in firmness was recorded in polyethylene glycol and 0.5% sodium alginate in diluted (1:4) carnauba wax emulsion (T2), while the maximum decrease (8.56 N–5.54 N and 8.56 N–3.57 N) was recorded in the control (T4) packaged and unpackaged eggplants after 12 days of storage, respectively [21].

### 2.6. Microbial Analysis

**Figure 7:**
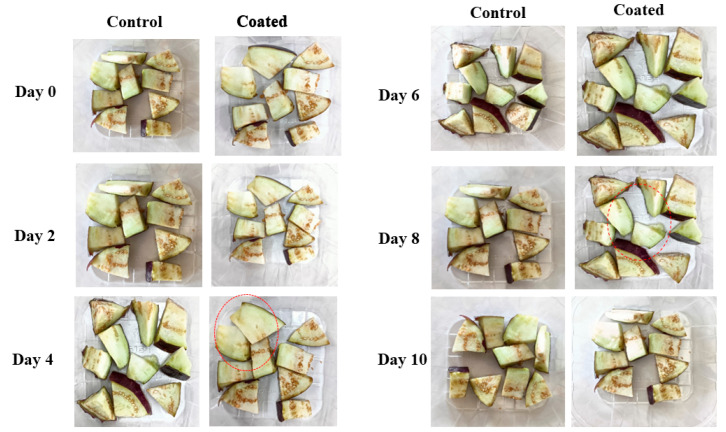
The visual appearance of TMCG-coated and uncoated cut brinjals.

**Figure 8:**
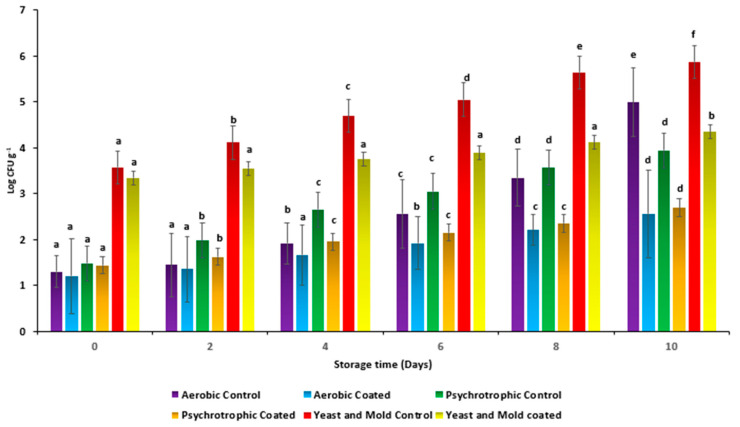
Antimicrobial efficacy of cut apples against bacteria and fungus.

Edible coating gel solution prepared from the TMCG showed a significant effect on the shelf life of cut brinjals by reducing microbial growth. Microbial growth of the control brinjals significantly increased during the storage period (10 days). Overall, AMCG coating maintained the freshness of the cut-fresh brinjals for 10 days (Figure 7). Moreover, psychotropic and aerobic bacterial counts for the coating-treated brinjals were significantly lower than the control sample. For uncoated brinjals, psychotropic and aerobic counts were found to be 1.48 ± 0.25 log CFU g^−1^ and 1.3 ± 0.68 log CFU g^−1^ on the 0th day, which significantly increased up to 3.34 ± 0.57 log CFU g^−1^ and 4.99 ± 0.38 log CFU g^−1^ on the 10th day, respectively, whereas, for the coated sample, it was 1.44 ± 0.69 log CFU g^−1^ and 1.20 ± 0.43 log CFU g^−1^ on the 0th day, which increased up to 2.69 ± 0.66 log CFU g^−1^ and 4.99 ± 0.21 log CFU g^−1^ on the 10th day, respectively. Herein, it can be observed that TM-coated brinjal has good quality and less microbial growth as compared with the control sample, because mucilage itself has remarkable microbial activity, as shown in Figure 8.

On the other hand, yeast and mold counts for the control samples were 3.57 ± 0.38 log CFU g^−1^ on the 0th day and 3.34 ± 0.68 log CFU g^−1^ for edible coated brinjals on the 0th day, whereas, on the 10th day, yeast and mold growth significantly increased up to 5.87 ± 0.88 log CFU g^−1^ for the control and 4.35 ± 0.75 log CFU g^−1^ for the coated brinjals, respectively. TM is a good source of uronic acid. These uronic acids have great efficacy against various food pathogenic microorganisms. Also, the gelling property of mucilage prevents the food from physical, chemical, and biological hazards, resulting in the enhancement of the shelf life of brinjals [15]. TM can inhibit the bacterial adhesion to the host tissues and cells by preventing the bacteria from adhering to surfaces; they hinder the initial steps of bacterial colonization and infection. Moreover, TM can interact with the bacterial cell membranes; they disturb the integrity of the bacterial cell membrane by binding to lipopolysaccharides or peptidoglycans, leading to the leakage of cellular contents and ultimately cell lysis. Overall, TM has antibacterial properties that can be attributed to its ability to increase cell membrane permeability, prevent pathogenic bacteria from adhering to host cells, or hinder the transmembrane movement of nutrients or energy molecules [23,24,25,26,27].

## 3. Conclusions

The formulation of TMCG solution showed excellent commercial features like reduced physiological weight loss and maintained the freshness of cut brinjals by preventing them against environmental factors for approximately up to 15 days. Further, much more research may be carried out to evaluate the antimicrobial and antifungal properties of these TM-coated eggplants with effectiveness on long storage stability, retention, and activeness of other useful physiochemical and bioactive compounds. The effectiveness of TMCG as a potential edible coating material extended the shelf life of cut brinjals and retained the antioxidant activity and other nutritional properties of brinjals. Consequently, it holds promise for application in various sectors, both within the food industry and beyond. However, there is presently a scarcity of literature on TM gel, which could be expanded to uncover new reservoirs of mucilage as a natural substitute for synthetic polymers. Scientists are vigorously exploring avenues to supplant artificial materials. In this context, TM shows the potential to mitigate crucial shortcomings on an industrial scale. It is a water-soluble, edible polysaccharide with a gel-like texture and desirable functional and biological activities. It is a natural, renewable resource that is biodegradable. This makes it an environmentally friendly alternative to synthetic additives or thickeners. It is cost effective, easily available, eco-friendly, and non-toxic. This can be an attractive feature for consumers looking for sustainable and eco-friendly options.

## 4. Materials and Methods

### 4.1. Materials

Fresh purple brinjals (eggplant) were harvested from the agriculture farm of the Lovely Professional University, Punjab, India. Medium-sized taro rhizomes were obtained from the Punjab Agricultural University (PAU), Punjab, India. Nutrient agar and potato dextrose agar were used for the microbial analysis. These were procured from Titan Biotech Ltd., Mumbai, Maharashtra, India. Chemicals including 2,2-diphenyl-1-picryl-hidrazil (DPPH), 2,6-dichlorophenolindophenol, and Folin–Ciocalteu reagent were procured from ACS chemicals, Chennai, India.

### 4.2. Sample Preparation

Fresh brinjals (eggplant) were washed with running tap water to remove the dust and other impurities and stored at refrigeration conditions of 4–7 °C for 24 h. During the experiment, the brinjals were first rinsed with double distilled water, followed by the peeling and cutting of them into similar pieces (cube shape), each measuring approximately 7 cm in length, 4 cm in width, and 2.2 cm in thickness, using a sharp stainless-steel knife. To minimize their contact with oxygen, a maximum of 10 eggplants were processed simultaneously, and the average weight of the brinjals was found to be 210.34 ± 4.55 g. This entire procedure was conducted in a temperature-controlled environment at 10 °C, ensuring that hygienic conditions were maintained throughout.

### 4.3. Extraction of TM

TM was extracted using hot-water extraction according to the method proposed by Andrade et al. [16] with some variation in the extraction condition. Herein, taro rhizomes (100 g) were peeled and cut into a similar size and shape. Cut rhizomes were added into 150 mL of distilled water for the preparation of a slurry using a laboratory blender (VWR’s laboratory blenders, Delhi, India) and the sample was placed on the heating mantle (Borosil GMC500 200W Digital) at 70 °C for 30 min. The prepared slurry was then filtered using muslin cloth to remove solid material. Further, the sample was centrifuged at 10,000 rpm for 15 min. The supernatant was collected and dried at 40 °C in a vacuum oven. The dried powder was stored in an airtight glass container. The yield of mucilage was calculated using Equation (1).

(1) $$Yield\left(\%\right)=\left[\left(\frac{Total\;weight\;of\;mucilage\;(dry\;basis)}{Total\;weight\;of\;rhizomes}\times 100\right)\right]$$

### 4.4. Formulations of Edible Coating Gel Solutions

Edible coating gel solutions were prepared according to a method described by Moreira et al. [28]. Different aqueous solutions of TM (1, 2, 3, 4, 5, 6, and 7% *w*/*v*) were prepared (Table 1) in deionized water with continuous stirring using a magnetic stirrer for 30 min at 70 °C. After cooling the solutions at room temperature (28 °C), the solutions were centrifuged at 6000 rpm for 15 min to obtain a clear solution. The smallest particle-sized solutions were used for further application. Cut brinjals (100 g) were dipped into the 100 mL coating gel solution for 4 min, whereas 100 g of cut brinjals were kept as the control (without coating) and stored at refrigeration conditions of 4–7 °C for 10 days. At every 2-day interval, 10 g of coated and uncoated cut brinjals was randomly selected for the physicochemical analysis.

### 4.5. Characterization of Coating Gel Solution

#### 4.5.1. Particle Size and Zeta Potential

The particle size and intensity distribution of the samples were determined using dynamic light scattering (DLS) with a Zeta Sizer Nano ZS instrument from Microtrac MRB’s NANOTRAC Wave II Instruments. To prevent multiple scattering effects during the analysis, all samples were initially diluted 2000-fold with double distilled water. The Z-average size of the droplets was subsequently recorded. The analysis was conducted with duplicate samples, and three measurements were taken to ensure the repeatability of the results.

#### 4.5.2. Rheological Behavior

The rheological properties of the TMCG solution were assessed using an Anton Paar, Rheometer, China. A rheometer equipped with a Peltier heating/cooling system and a parallel plate layout was used for the experiment. For the evaluation of the rheological behavior, the coating gel solution was maintained at 50 °C for 40 min and the following pre-shearing procedure was applied to the solution prepared under various conditions: pre-shearing at a shear rate of 5 s^−1^ for 10 min, gradually increasing the shear rate from 3 to 40 s^−1^ over 4 min. A constant shear rate of 40 s^−1^ for 2 min was maintained with a reduction in the shear rate back to 3 s^−1^.

#### 4.5.3. Determination of Functional Groups

Fourier transform infrared spectroscopy (FTIR) was employed to determine the functional groups within the TMCG solution. The prepared coating gel solution was subjected to drying in a tray dryer at 60 °C for 12 h, following which an assessment of the functional groups was carried out. FTIR analysis was conducted using a Perkin Elmer instrument, which utilized infrared radiation in the mid-region spanning from 4000 to 400 cm^−u^ to determine the presence of active functional groups within the polysaccharide coating gel solution.

#### 4.5.4. Scanning Electron Microscopy (SEM) of TMCG

The surface morphology and shape of the TMCG were examined using a scanning electron microscope (JSM5910, JEOL, Tokyo, Japan). The resulting TMCG formulation was applied using dual-sided adhesive tape to metal stubs in order to facilitate SEM measurements. It was vacuum chambered, sputter coated with a 10 nm thick layer of gold and examined at 10,000 magnification using a high-resolution scanning electron microscope.

### 4.6. Application of the Coating Gel Solution of Cut Brinjals

#### 4.6.1. Weight Loss and pH Value of Cut Brinjals

Weight loss of coated and uncoated cut brinjals was assessed by following a method described by Le et al. [29]. Briefly, cut coated and uncoated brinjals were placed into pre-weighted plastic trays and stored under controlled refrigeration conditions. Weight loss was assessed for the 10 days of the storage period at different time intervals and all experiments were conducted in triplicate. Additionally, the pH of the brinjals was studied, following the methodology outlined by Panahirad et al. [30], at different intervals such as 0th, 2nd, 4th, 6th, 8th, and 10th. To analyze the pH, 10 g of cut brinjals (coated and uncoated) were homogenized in 30 mL of deionized water using a high-speed homogenizer, and the pH was determined using a digital pH meter (Forbes Marshall, Mumbai, India) for both samples.

#### 4.6.2. TSS and Acidity of Cut Brinjals

The total soluble solids (TSS) and acidity of the coated and uncoated cut brinjals were assessed based on the method outlined by Abbasi et al. [31]. The procedure involved homogenizing the brinjals (20 g) using a laboratory hand blender (Fisher Scientific, Surat, India), with the addition of 50 mL distilled water to prepare a slurry. This slurry was then utilized to evaluate both the titratable acidity and TSS. To determine TSS, the resulting slurry was filtered using Whatman filter paper 1, and the filtrate was further purified through centrifugation at 6000× *g* for 10 min. A digital reflectometer (Techno Instrumentation Pvt. Ltd., Chennai, India) was used to measure the TSS of the brinjals. For titratable acidity, the brinjal juice slurry was titrated with a 0.1 M NaOH solution, employing phenolphthalein as an indicator to detect the endpoint of the titration.

#### 4.6.3. Antioxidant Activity of Cut Brinjals

The antioxidant activity of the control and coated brinjals was determined following the method recommended by de Souza et al. [32] using the 2,2-diphenyl-1-picryl-hydrazyl (DPPH) radical scavenging assay. The assessment involved measuring the absorbance of the samples using a spectrophotometer at a wavelength of 517 nm. The antioxidant activity was quantified using the following Equation (2) and expressed as the percentage of DPPH radical inhibition:

(2) $$Antioxidant\;activity\left(\%\right)=\left[\left(\frac{Absorbance\;of\;sample-Absorbance\;of\;control}{Absorbance\;of\;sample}\times 100\right)\right]$$

#### 4.6.4. Ascorbic Acid Content of Cut Brinjals

The ascorbic acid (vitamin C) content of coated and control cut brinjals was determined following the methodology outlined by Saleem et al. [33]. The analysis involved titration with 2,6-dichlorophenolindophenol (DCPIP) to measure the concentration of ascorbic acid. The results were expressed in mg of vitamin C per 100 g of cut apples. To establish a standard curve, various concentrations of ascorbic acid were utilized as reference points for comparison during titration. This allowed for the precise quantification of ascorbic acid content in the brinjal samples.

#### 4.6.5. Firmness

The firmness of edible coated and uncoated cut brinjals was analyzed according to the method suggested by Huang et al. [34]. A texture analyzer (AMETEK Brookfield CT3, Beijing, China) was used for the determination of firmness. This analysis involved using a 7 mm diameter probe, which applied a higher penetration force to penetrate a 20 mm tall cut brinjal piece to a depth of 12 mm at a constant rate of 15 mm/s. For the experiment, four randomly selected cut brinjals (two coated pieces and two uncoated) were chosen on the 0th, 2nd, 4th, 6th, 8th, and 10th days. These pieces were positioned perpendicular to the probe to allow for penetration at their geometric center, thus measuring their firmness.

### 4.7. Microbial Analysis

Microbiological analysis, for the determination of yeast and mold counts, total psychrotrophic counts, and aerobic plate counts, was conducted in triplicate for both the uncoated and coated sliced brinjals at various time intervals (0, 2, 4, 6, 8, and 10 days). The procedure followed was in accordance with the methodology outlined by Paolucci et al. [35]. Fresh-cut brinjals were taken for this analysis. To summarize, 30 g samples of sliced brinjals per treatment was homogenized in sterile stomacher bags. Subsequently, 20 g of the homogenized brinjals was transferred to another stomacher bag and mixed with 70 mL of buffered peptone water to ensure uniform distribution. The mixture was homogenized for 4 min followed by 10-fold dilutions prepared using this diluent, and specific Petri films were used for microbial counts, with 3M aerobic plate count (APC) plates for aerobic counts and 3M yeast and mold count plates for yeast and mold counts. The 3M APC plates were incubated at 37 °C for 48 h to determine aerobic counts. For psychrotrophic counts, the APC plates were incubated at 4 °C for 5 days, while all 3M yeast and mold count plates were incubated at 20 °C for 6 days. After incubation, colonies were counted, and the results were expressed as log CFU (colony-forming units) per gram of the sample. These data provide valuable insights into the microbial quality and safety of the brinjals during their storage.

### 4.8. Statistical Analysis

All the experimental data performed in this study are represented in the format of average mean ± standard deviation. For statistical analysis, Microsoft Excel 2022 and SPSS software (version 17 by SPSS Inc.) were used to calculate all investigational results. Each experiment was conducted in triplicate to ensure data consistency and to minimize the impact of random variability. One-way ANOVA was applied to assess the significance of differences among the samples.

## Figures and Tables

**Table 1:** Different formulations, average particle size, and zeta potential of edible coating gel solution.

Treatment	Concentration (%)	Average Particle Size (nm)	Zeta Potential (mV)
T1	1	224 ± 0.68	−24.18 ± 0.44
T2	2	204 ± 0.94	−16.14 ± 0.67
T3	3	189 ± 0.73	−31.87 ± 0.35
T4	4	171 ± 0.55	−11.27 ± 0.27
T5	5	154 ± 0.81	−27.22 ± 0.75
T6	6	166 ± 1.68	−36.46 ± 0.34
T7	7	187 ± 0.47	−20.67 ± 0.66

## Data Availability

The data are contained within the article.

## References

[1] Manthei A., López-Gámez G., Martín-Belloso O., Elez-Martínez P., Soliva-Fortuny R. (2023). Relationship between Physicochemical, Techno-Functional and Health-Promoting Properties of Fiber-Rich Fruit and Vegetable By-Products and Their Enhancement by Emerging Technologies. Foods.

[2] Khan U.M., Sameen A., Aadil R.M., Shahid M., Sezen S., Zarrabi A., Ozdemir B., Sevindik M., Kaplan D.N., Selamoglu Z. (2021). Citrus genus and its waste utilization: A review on health-promoting activities and industrial application. Evid.-Based Complement. Altern. Med..

[3] Farooq M., Azadfar E., Rusu A., Trif M., Poushi M.K., Wang Y. (2021). Improving the Shelf Life of Peeled Fresh Almond Kernels by Edible Coating with Mastic Gum. Coatings.

[4] Ghag S.S., Gokhale J.S., Lele S.S. (2023). Effect of chemical pretreatment on quality attributes of the cashew apple. J. Food Sci..

[5] Lyn F.H., Adilah Z.M., Nor-Khaizura M.A.R., Jamilah B., Hanani Z.N. (2020). Application of modified atmosphere and active packaging for oyster mushroom (*Pleurotus ostreatus*). Food Packag. Shelf Life.

[6] Kalpana S., Priyadarshini S.R., Leena M.M., Moses J.A., Anandharamakrishnan C. (2019). Intelligent packaging: Trends and applications in food systems. Trends Food Sci. Technol..

[7] Fiore A., Park S., Volpe S., Torrieri E., Masi P. (2021). Active packaging based on PLA and chitosan-caseinate enriched rosemary essential oil coating for fresh minced chicken breast application. Food Packag. Shelf Life.

[8] Goksen G., Demir D., Dhama K., Kumar M., Shao P., Xie F., Echegaray N., Lorenzo J.M. (2023). Mucilage polysaccharide as a plant secretion: Potential trends in food and biomedical applications. Int. J. Biol. Macromol..

[9] Kaith B.S., Singh A., Sud D. (2022). A new horizon for structural modifications and comprehensive applications of *Colocasia esculenta* (L.). Schott. Mater. Today Proc..

[10] Hendek Ertop M., Atasoy R., Akın Ş.S. (2019). Evaluation of taro [*Colocasia Esculenta* (L.) Schott] flour as a hydrocolloid on the physicochemical, rheological, and sensorial properties of milk pudding. J. Food Process. Preserv..

[11] Tosif M.M., Najda A., Klepacka J., Bains A., Chawla P., Kumar A., Sharma M., Sridhar K., Gautam S.P., Kaushik R. (2022). A concise review on taro mucilage: Extraction techniques, chemical composition, characterization, applications, and health attributes. Polymers.

[12] Biswas A., Ahmed T., Rana M.R., Hoque M.M., Ahmed M.F., Sharma M., Sridhar K., Ara R., Stephen Inbaraj B. (2023). Fabrication and Characterization of ZnO Nanoparticles-Based Biocomposite Films Prepared Using Carboxymethyl Cellulose, Taro Mucilage, and Black Cumin Seed Oil for Evaluation of Antioxidant and Antimicrobial Activities. Agronomy.

[13] Farousha K., Rangaraj V.M., Rambabu K., Haija M.A., Banat F. (2023). Development of date seed extract encapsulated MCM-41: Characterization, release kinetics, antioxidant and antibacterial studies. Food Biosci..

[14] Krishnamoorthy R., Hai A., Banat F. (2023). Subcritical Water Extraction of Mango Seed Kernels and Its Application for Cow Ghee Preservation. Processes.

[15] Safdar B., Pang Z., Liu X., Jatoi M.A., Rashid M.T. (2022). Rheological and tribological nature of flaxseed gum influenced by concentration and temperature and its application as a coating agent for potato chips. J. Food Sci..

[16] Andrade L.A., de Oliveira Silva D.A., Nunes C.A., Pereira J. (2020). Experimental techniques for the extraction of taro mucilage with enhanced emulsifier properties using chemical characterization. Food Chem..

[17] Thakur S., Bains A., Sridhar K., Kaushik R., Gupta V.K., Chawla P., Sharma M. (2023). Gum Arabic/guar gum based biopolymeric nanohydrogel for shelf-life enhancement of grapes and photocatalytic dye reduction. Ind. Crops Prod..

[18] Tosif M.M., Bains A., Dhull S.B., Chawla P., Goksen G. (2023). Effect of Aloe vera and carboxymethyl cellulose-derived binary blend edible coating on the shelf life of fresh-cut apple. Food Sci. Nutr..

[19] Amanullah S., Jahangir M.M., Ikram R.M., Sajid M., Abbas F., Mallano A.I. (2016). Aloe vera coating efficiency on shelf life of eggplants at differential storage temperatures. J. Northeast. Agric. Univ. (Engl. Ed.).

[20] Anjum M.A., Akram H., Zaidi M., Ali S. (2020). Effect of gum arabic and Aloe vera gel based edible coatings in combination with plant extracts on postharvest quality and storability of ‘Gola’guava fruits. Sci. Hortic..

[21] Singh S., Singh B., Alam T. (2019). Evaluation of shelf-life, antioxidant activity and nutritional quality attributes in carnauba wax coated eggplant genotypes. J. Food Sci. Technol..

[22] Rangaraj V.M., Devaraju S., Rambabu K., Banat F., Mittal V. (2022). Silver-sepiolite (Ag–Sep) hybrid reinforced active gelatin/date waste extract (DSWE) blend composite films for food packaging application. Food Chem..

[23] Ali S.S., Ahsan H., Zia M.K., Siddiqui T., Khan F.H. (2020). Understanding oxidants and antioxidants: Classical team with new players. J. Food Biochem..

[24] Miteluț A.C., Popa E.E., Drăghici M.C., Popescu P.A., Popa V.I., Bujor O.C., Ion V.A., Popa M.E. (2021). Latest developments in edible coatings on minimally processed fruits and vegetables: A review. Foods.

[25] Hassoun A., Cropotova J., Trif M., Rusu A.V., Bobiş O., Nayik G.A., Jagdale Y.D., Saeed F., Afzaal M., Mostashari P. (2022). Consumer acceptance of new food trends resulting from the fourth industrial revolution technologies: A narrative review of literature and future perspectives. Front. Nutr..

[26] Iqbal M.W., Riaz T., Yasmin I., Leghari A.A., Amin S., Bilal M., Qi X. (2021). Chitosan-based materials as edible coating of cheese: A review. Starch-Stärke.

[27] Shanta S.S., Ahmed T., Jubayer M.F., Sharma M., Sridhar K., Hoque M.M., Rana M.R., Inbaraj B.S. (2023). Effect of Taro Corm Mucilage and Black Seed Oil as Edible Coatings on the Shelf-Life and Quality of Fresh Guava. Agronomy.

[28] Moreira B.R., Pereira-Junior M.A., Fernandes K.F., Batista K.A. (2020). An ecofriendly edible coating using cashew gum polysaccharide and polyvinyl alcohol. Food Biosci..

[29] Le K.H., Nguyen M.D.B., Dai Tran L., Thi H.P.N., Van Tran C., Van Tran K., Thi H.P.N., Thi N.D., Yoon Y.S., Nguyen D.D. (2021). A novel antimicrobial ZnO nanoparticles-added polysaccharide edible coating for the preservation of postharvest avocado under ambient conditions. Prog. Org. Coat..

[30] Panahirad S., Naghshiband-Hassani R., Bergin S., Katam R., Mahna N. (2020). Improvement of postharvest quality of plum (*Prunus domestica* L.) using polysaccharide-based edible coatings. Plants.

[31] Abbasi A., Sabahi S., Bazzaz S., Tajani A.G., Lahouty M., Aslani R., Hosseini H. (2023). An edible coating utilizing Malva sylvestris seed polysaccharide mucilage and postbiotic from Saccharomyces cerevisiae var. boulardii for the preservation of lamb meat. Int. J. Biol. Macromol..

[32] de Souza E.L., Lundgren G.A., de Oliveira K.Á., Berger L.R., Magnani M. (2019). An analysis of the published literature on the effects of edible coatings formed by polysaccharides and essential oils on postharvest microbial control and overall quality of fruit. Compr. Rev. Food Sci. Food Saf..

[33] Saleem M.S., Anjum M.A., Naz S., Ali S., Hussain S., Azam M., Sardar H., Khaliq G., Canan İ., Ejaz S. (2021). Incorporation of ascorbic acid in chitosan-based edible coating improves postharvest quality and storability of strawberry fruits. Int. J. Biol. Macromol..

[34] Huang P., Ding J., Liu C., Li H., Wang C., Lin Y., Sameen D.E., Hossen M.A., Chen M., Yan J. (2023). Konjac glucomannan/low-acyl gellan gum edible coating containing thymol microcapsule regulates cell wall polysaccharides disassembly and delays postharvest softening of blueberries. Postharvest Biol. Technol..

[35] Paolucci M., Di Stasio M., Sorrentino A., La Cara F., Volpe M.G. (2020). Active edible polysaccharide-based coating for preservation of fresh figs (*Ficus carica* L.). Foods.

